# Abscisic Acid and Glycine Betaine Mediated Tolerance Mechanisms under Drought Stress and Recovery in *Axonopus compressus*: A New Insight

**DOI:** 10.1038/s41598-020-63447-0

**Published:** 2020-04-24

**Authors:** Mohsin Nawaz, Zhiyong Wang

**Affiliations:** Key Laboratory of Genetics and Germplasm Innovation of Tropical Special Forest Trees and Ornamental Plants, Ministry of Education, College of Forestry, Hainan University, Haikou, 570228 P.R. China

**Keywords:** Systems biology, Climate-change ecology, Plant sciences, Plant physiology, Plant stress responses

## Abstract

Changing climatic scenarios affect plant growth and consequences are more malicious in drought conditions. This study was performed for better understanding of tolerance mechanisms under prevailing drought stress and succeeding recovery in *Axonopus compressus* by exogenously applied abscisic acid (ABA) and glycine betaine (GB). Three *A. compressus* accessions (A-38, A-58 and A-59) were subjected to well-watered (100% field capacity) and drought (40% field capacity) conditions. Two weeks later, plants were recovered from drought by re-watering. Water (control), GB, ABA and their combination were foliar applied on plants under drought twice a week until recovery. Drought stress decreased photosynthetic pigments and increased reactive oxygen species, lipid peroxidation, osmolytes and antioxidants in all accessions of *A. compressus*. Nonetheless, exogenous ABA and GB alone or in combination improved drought tolerance in all accessions which was maintained even after recovery. Maximum decrease in hydrogen peroxide and malondialdehyde, and increase in soluble sugars, proteins, proline, phenolics and chlorophyll contents, and superoxide dismutase, catalase, peroxidase and ascorbate peroxidase activity was recorded when GB was applied alone under drought. Order of improvement in drought tolerance among accessions was A-58 > A-59 > A-38. In conclusion, improved drought tolerance mechanisms by ABA and GB in *A. compressus* were retained even after recovery.

## Introduction

Plants experience recurrent drought conditions which is one of the major negative forces for growth and productivity of biological systems^1,2^. Drought stress is one of the key ecological effectors which causes alterations at morpho-physiological, biochemical and molecular levels^3^. Water deficit reduces cell division and elongation, disturbs tissue water status, causes stomatal closure, damages photosynthetic machinery, limits the photosynthesis, and hampers nutrient uptake and translocation ultimately affecting plant growth and productivity^3–5^. In addition, drought stress enhances production of reactive oxygen species (ROS) *viz*. singlet oxygen (O^*^), hydroxyl radicals (OH ∙ .), superoxide (O_2_^−^) and hydrogen peroxide (H_2_O_2_)^6,7^, which causes exaggerated lipid peroxidation of cellular membranes^7,8^, denaturation of proteins, destruction of nucleic acid ultimately disrupting homeostasis^6,9,10^.

Plants have devised tolerance mechanisms which potentially promote their survival in drought stressed environments^11,12^. Abiotic stress induced ROS production activates the signaling mechanisms in plants^8,13,14^, which induce hormonal modifications mainly increase in endogenous ABA, and activates the genes for production of osmolytes and antioxidant enzymes^3,15–17^. Compatible solutes such as proline, glycine betaine, soluble proteins ad sugars, organic acids and phenolic compounds are accumulated in plants in response to drought which improve the water potential, detoxify ROS, and protect cellular membranes and macromolecules from lipid peroxidation^11,18,19^. In addition, activity of antioxidants including both enzymatic *viz*. superoxide dismutase (SOD), catalase (CAT), peroxidase (POD), glutathione reductase (GR) and ascorbate peroxidase (APX) as well as non-enzymatic *viz*. ascorbic acid (AsA) and glutathione (GS) is exalted under drought stress which scavenge the ROS and protect biological membranes and organic molecules^18,20^. Previous studies have reported that tolerant cultivars had greater osmolytes and antioxidants activity under drought stress^18,21^.

Plant growth regulating substances have the potential to improve stress tolerance mechanisms in plants. Previous studies showed that exogenous application of growth regulators and osmolytes considerably reduced the oxidative damage in plants under drought stress^20,22,23^. Application of ABA under stressed conditions have proved beneficial in improving the stress tolerance mechanisms in plants. Abscisic acid concentration is increased in plant cells in response to drought stress which affects stomatal closure, initiates the cascade signaling and activates the defense mechanisms such as elevation of antioxidants activity^3,11^. Exogenous ABA remarkably enhances net photosynthesis, stomatal conductance and production of primary and secondary metabolites such as phenolics and flavonoids in plants under stressful condition^19,24–26^. Similarly, it has been noticed that the capacity of antioxidant defense systems, both enzymatically and non-enzymatically, can be improved by the ABA application under drought^27^. Previous studies have reported that exogenous ABA application enhanced the ambulation of proline, and activity of guaiacol peroxidase, CAT, SOD and APX activities^27–29^.

Glycine betaine acts as compatible solute and helps in improving the tolerance in plants against abiotic stresses by improving the tissue water status and protecting the biological membranes from ROS under drought stress^9,30^. Similarly, the plant defensive mechanisms including osmotic balance, enzymes, and genes associated with resistance have also been well documented to be enhanced by the GB^16,31,32^. Moreover, GB have been found to improve the photosynthesis under drought stress by improving the Ca^2+^-ATPase and Hill reaction activities in thylakoid membrane system^33,34^. Previous studies have reported that GB improved drought tolerance in sunflower^35^, cumin^36^ and wheat^37^.

Under the scenario of abrupt changes in climatic conditions, there is a dire need to find the way of reducing the disastrous effects of the limited water condition. There are many instances where the abiotic stress is accompanied by recovery and subsequent stress which affect the plant growth and development. In such circumstances it is crucial to retain the stress tolerance mechanisms active during the recovery period until the recurrent stress occurs in order to keep the plant growth in pace. However, studies on exogenously applied growth substances for improving the stress tolerance in plants under drought and sustenance of tolerance mechanisms active during subsequent recovery period are scant. *Axonopus compressus* L. is commonly known as carpet grass and belongs to Poaceae family. It is commonly used for groundcover, turf in low fertility soils, and permanent pasture. In this study it was hypothesized that exogenous application of ABA and GB will improve drought tolerance in *A. compressus* and retain tolerance mechanisms active following recovery. This study was conducted with objectives to determine the effect of exogenous application of ABA and GB alone or in combination on photosynthetic pigments, ROS production and lipid peroxidation, osmolytes accumulation and antioxidants activity in *Axonopus compressus* accessions under drought stress and recovery.

## Material and Method

### Plant culture

The experiment was carried out in glass house at Experimental Station of Institute of Tropical Agriculture and Forestry, Hainan University (20° 03′ 22.80″N, 110° 19′ 10.20″E), China during 2018. Three accession of *A. compressus* (A-58, A-38 and A-59) acquired from the germplasm resource library maintained at the Hainan University, were used as experimental material. The accessions were selected on the basis their tolerance to drought stress i.e. A-38 (sensitive), A-59 (moderately tolerant) and A-58 (tolerant). These accessions show apparently similar phenology and quality potentials under the conducive growth conditions. The cuttings of uniform size were selected for propagation and surface-sterilized by dipping in 0.5% Na-hypochlorite for five min followed by thorough washing with double distilled water (ddH_2_O). Five cuttings were planted in each soil filled plastic pot (27 cm in diameter, 17 cm in depth) with freely draining irrigation water. The average night/day temperature (T) in the glass house was 26–31 °C and relative humidity (RH) was 70% during the growth period.

### Imposition of drought stress

After 25-days of growth under normal conditions, the uniform seedlings in pots were retained for subsequent study. Two levels of drought stress were imposed *viz*. well-watered (100% field capacity) and drought stress (40% field capacity). Drought stress was imposed by skipping the irrigation until the field capacity of 40% was maintained. Desired levels of soil moisture were acquired by determining the amount of water required for attaining specified field capacity, weighing pots after watering with calculated amount of water and designating it as target weight. Water was applied every alternate day to attain the target pot weight for imposing drought stress. Two weeks after drought imposition the pots were brought to the well-watered condition by re-watering for recovery from drought stress.

### ABA and GB treatments and plant sampling

**Figure 1:**
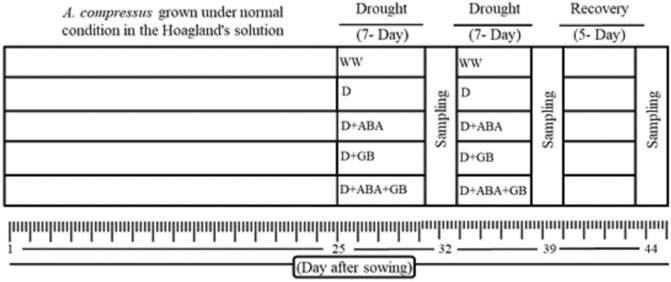
Experimental design of the study: drought stress treatments were applied after the 25 days of normal growth by withholding irrigation. The drought treatments were maintained for 14 days by weighing the pots and compensating the water lost to the desired FC and then followed by re-watering. The sampling were done after the one week and two week after the drought application and 5 day after rewatering. Whereas the WW; well-watered, D; drought, D + ABA; drought + abscisc acid, D + GB; drought + glycine betaine and D + ABA + GB; drought+ abscisic acid+ glycine betaine.

There were five sets of treatments each replicated three times, which include well-watered, drought stress, drought plus ABA, drought plus GB, drought plus ABA plus GB as described in the Fig. 1. The ABA and GB alone or in combination were applied twice a week after imposition of drought stress until recovery from drought stress. The knapsack sprayer was used to spray the plants with ABA and GB. Spray was performed on plant leaves until the spray solution drips down from the leaves. The same amount of water was sprayed on control plants. The concentration of 100 µmol for each of ABA and GB alone or in combination was used for spray. The treatment of ABA plus GB was applied by spraying them separately in order to avoid the chemical reaction between ABA and GB.

### Measurements

Measurements for physio-biochemical attributes were made one week after drought (1-WAD), two weeks after drought (2-WAD) and five days after re-watering (5-DARW). After leaving the top three uppermost leaves, fully expanded *A. compresuss* leaves from each replication were detached 1-WAD, 2-WAD and 5-DARW. Leaves were immediately stored in liquid-N after detachment to instantaneously stop the biological activities in sampled leaves and then stored at −70 °C in refrigerator for determining the hydrogen peroxide (H_2_O_2_), malondialdehyde (MDA), osmolytes and antioxidant enzymes. However, photosynthetic pigments, electrolyte leackage (EL) and membrane stability index (MSI) was determined from freshly detached leaves.

### Hydrogen peroxide and malondialdehyde contents

The H_2_O_2_ content in fresh leaf samples of *A. compresuss* was determined by homogenizing in ice-bath with trichloroacetic acid (TCA), centrifuging and adding the supernatant to K-phosphate buffer and K-iodide. The absorbance of reaction mixture was read by using MPDA-1800 (Shanghai- China) spectrophotometer. The H_2_O_2_ was calculated against standard curve^38^. For determination of MDA, the leaves samples were homogenized in TCA solution, centrifuged and mixed with thiobarbituric acid (TBA) solution. The MDA concentration was assayed by reading absorbance of supernatant with spectrophotometer according to^39^.

### Electrolyte leakage and photosynthetic pigments

Freshly collected leaf disks were soaked in distilled water at room temperature for six hours in a shaker and electrical conductivity was determined using a conductivity meter (Mettler-Toledo Co., Ltd, Shanghai, China). Afterwards; samples were heated in water bath in boiling water for 30 min and electrical conductivity was measured. The electrolyte leakage was determined by using formula EL = (EC1/EC2) × 100 as described by Anjum *et al*.^40^. Chlorophyll pigments extraction was done in acetone and estimated by MPDA-1800 spectrometer (Shanghai- China). Chlorophyll (Chl) *a*, *b*, *a* + *b* and carotenoid contents were estimated according to Lichtenthaler^41^.

### Osmolytes accumulation

Proline content in *A. compressus* leaves was determined by homogenizing the samples in sulfosalicylic acid and glacial acetic acid. It was followed by addition of ninhydrin solution in the filtrate, incubation and cooling in ice bath. Afterwards toluene was added in the mixture and vortexed. Red chromophore containing toluene was aspirated from mixture and proline was determined against standard curve according to^42^. Total soluble proteins in *A. compressus* leaves were assayed by extracting in the phosphate buffer saline. Total soluble proteins content was determined against standard curve prepared by using bovine serum albumin according to^43^. Soluble sugars content was determined by boiling the *A. compressus* leaves in distilled water in water bath at 100 °C for 30 min, cooling at room temperature and mixing. The soluble sugars content in the filtrate was determined by adding the anthrone-sulphuric acid reagent and raeding the absorbance according to the method of Zhang *et al*.^44^.

### Enzymatic antioxidant activities

Leaves samples of *A. compressus* were homogenized in potassium phosphate buffer containing ethylenediamine tetraacetic acid (EDTA) and PVP, centrifuged and supernatants were used as crude extracts for following antioxidant assays^45^. The SOD activities were measured spectrophotometrically by tracking the hang-up of the photochemical reduction of nitroblue tetrazolium (NBT)^46^. The reaction mixture containing sample extract, K-phosphate buffer, riboflavin, NBT, methionine and ethylenediamine tetraacetic acid (EDTA) was illuminated at a light intensity of 5 000 lx light intensity. One unit SOD activity was defined as the required amount of enzyme to upto 50% inhibition in reduction of NBT. The guaiacol method as advised by Upadhyaya^47^ was used to determine the activity of peroxidase (POD). The absorbance of reactions mixture having sample extract, K-phosphate buffer, H_2_O_2_ and guaiacol was read by using spectrophotometer to determine the POD activity. Catalase (CAT) activity was determined by measuring the rate of H_2_O_2_ decomposition at 240 nm absorbance in the reaction mixture comprised of Na- phosphate buffer, H_2_O_2_ and sample extract according to Aebi^48^. Ascorbate peroxidase assay was carried out by adding the supernatant to K-phosphate buffer, ASC and H_2_O_2_. The APX activity was determined by using the method of Nakano and Asada^49^.

### Experimental design and statistical analysis

The experiment was laid out using completely randomized design (CRD) having three replications. The data were analyzed by using statistical software ‘Statistix 8’ (Analytical software, Tallahassee, Florida, USA) for windows. A two-way analysis of variance (ANOVA) was used to test the difference among treatments and their interactions for different stages of sampling separately for each parameter. The difference amongst treatments’ means was determined by using least significant difference (LSD) test at 5% probability level.

## Results

### ROS production, lipid peroxidation and cell membrane stability

**Figure 2:**
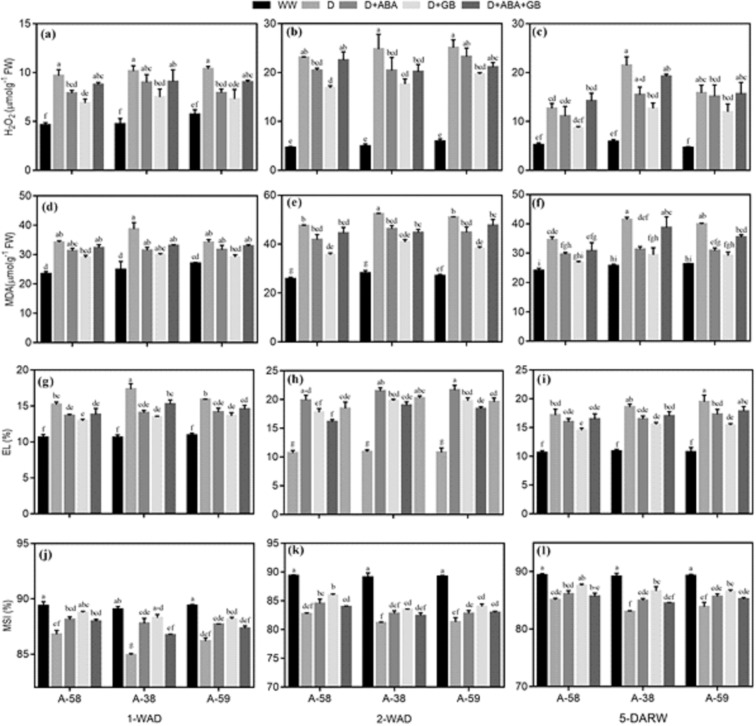
Effect of drought stress and subsequent re-watering on H_2_O_2_ (**a**–**c**), MDA (**d**–**f**) electrolyte leakage (EL) (**g**–**i**) and membrane stability index (MSI) (**j**–**l**) in A-58, A-38 and A-59 accessions of *A. compressus*. The measurements were done at one week after drought (1-WAD), two weeks after drought (2-WAD) and five days after re-watering (5-DARW). Bars are mean ± SE of three replications (n = 3). The bars having the same letters don’t differ significantly at p ≤ 0.05.

Drought stress substantially increased H_2_O_2_, MDA and EL while decreased MSI in all accessions of *A. compressus* and similar trend was maintained after recovery from drought, as compared to well-watered. Among accessions the order for H_2_O_2_, MDA and EL was A-38 > A-59 > A-58, while for MSI was A-58 > A-59 > A-38 at all stages. Highest H_2_O_2,_ MDA and EL, and lowest MSI was recorded at 2-WAD. However, re-watering started declining H_2_O_2,_ MDA and EL, and increasing MSI in all accessions of *A. compressus*. Exogenous application of ABA and GB alone or in combination decreased the H_2_O_2_, MDA and EL, and increased MSI in all accessions of *A. compressus* at all stages, as compared to control; while, application of GB alone proved most effective. Glycine betaine decreased H_2_O_2_, MDA and EL in A-59 (27–29%, 15–25% and 15–19%), A-38 (26–29%, 22–23% and 12–22%) and A-59 (22–30%, 15–25% and 14–15%), respectively, during drought stress as compared to untreated control. However, after re-watering the GB treatment decreased H_2_O_2_ (31%, 41% and 25%), MDA (27%, 29% and 27%) and EL (22%, 16% and 21%) in drought stressed plants of A-58, A38 and A-59, respectively. On the other hand, the MSI was enhanced by 2–4% and 3–4% due to GB application in *A. compressus* accessions during drought stress and subsequent recovery, respectively (Fig. 2).

### Photosynthetic pigments

**Figure 3:**
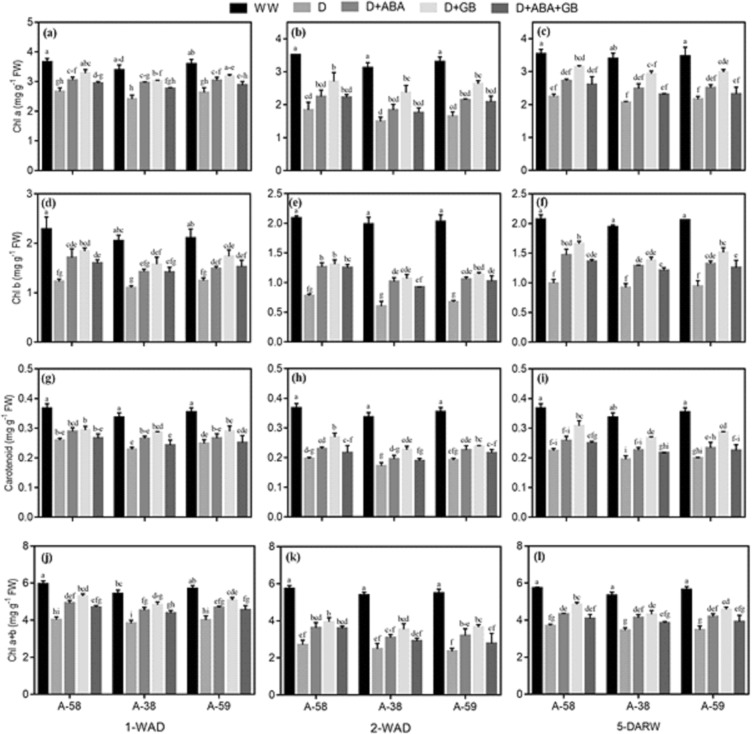
Effect of drought stress and subsequent re-watering on chlorophyl a (Chl a) (**a**–**c**), chlorophyl b (Chl b) (**d**–**f**), carotenoids (**g**–**i**) and chlorophyl a + b (Chl a + b) (**j**–**l**) in A-58, A-38 and A-59 accessions of *A. compressus*. The measurements were done at one week after drought (1-WAD), two weeks after drought (2-WAD) and five days after re-watering (5-DARW). Bars are mean ± SE of three replications (n = 3). The bars having the same letters don’t differ significantly at p ≤ 0.05.

The biosynthesis of photosynthetic pigments was reduced due to drought stress in all accessions of *A. compressus* at all stages, as compared to well-watered plants. However, the drought induced damages on photosynthetic pigments were more pronounced in A-38 while A-59 was relatively tolerant at all stages. Moreover, the deleterious effects of drought stress on photosynthetic pigments were increased with duration after exposure to drought (2-WAD) and decreased after recovery from drought due to re-watering (5-DARW). Treatment with ABA and GB alone or in combination improved drought tolerance in all accessions of *A. compressus* as indicated by enhanced Chl *a*, Chl *b*, Chl *a* + *b* and carotenoids contents, as compared to control. The effect of ABA and GB on photosynthetic pigments was persisted even after recovery from drought stress. Plants of A-58, A-38 and A-59 treated with GB alone exhibited 23–91%, 26–101% and 21–114% increase in photosynthetic pigments during drought stress and 40–87%, 42–101% and 38–101% increase 5-DARW, respectively (Fig. 3).

### Osmolytes accumulation

**Figure 4:**
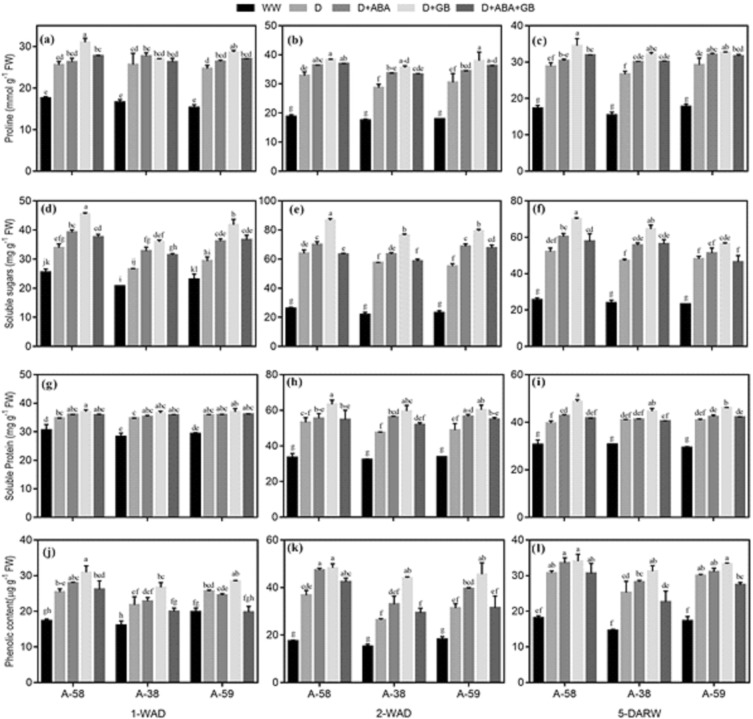
Effect of drought stress and subsequent re-watering on proline (**a**–**c**), soluble sugars (**d**–**f**), soluble proteins (**g**–**i**) and total soluble phenolics contents (**j**–**l**) in A-58, A-38 and A-59 accessions of *A. compressus*. The measurements were done at one week after drought (1-WAD), two weeks after drought (2-WAD) and five days after re-watering (5-DARW). Bars are mean ± SE of three replications (n = 3). The bars having the same letters don’t differ significantly at p ≤ 0.05.

Production and accumulation of proline, soluble sugars, proteins and phenolics was triggered by drought stress in all accessions of *A. compressus* at all stages of drought and recovery, as compared to well-watered conditions. Among accessions drought stressed plants of A-58 accumulated the greatest quantity of osmolytes while A-38 exhibited least accumulation during drought (1-WAD and 2-WAD) and after recovery (5-DARW), as compared to well-watered plants. Maximum accumulation of osmolytes occurred at 2-WAD; however, the osmolytes accumulation was decreased again 5-DARW. Treatment with ABA and GB further exacerbated the osmolytes accumulation in *A. compressus* accessions under drought stress and after recovery, as compared to untreated control. Application of GB alone exalted accumulation of osmolytes by 6–35%, 5–67% and 3–45% under drought stress, and 11–34%, 6–37% and 9–17% after re-watering in A-58, A-38 and A-59, respectively (Fig. 4).

### Activity of antioxidant enzymes

**Figure 5:**
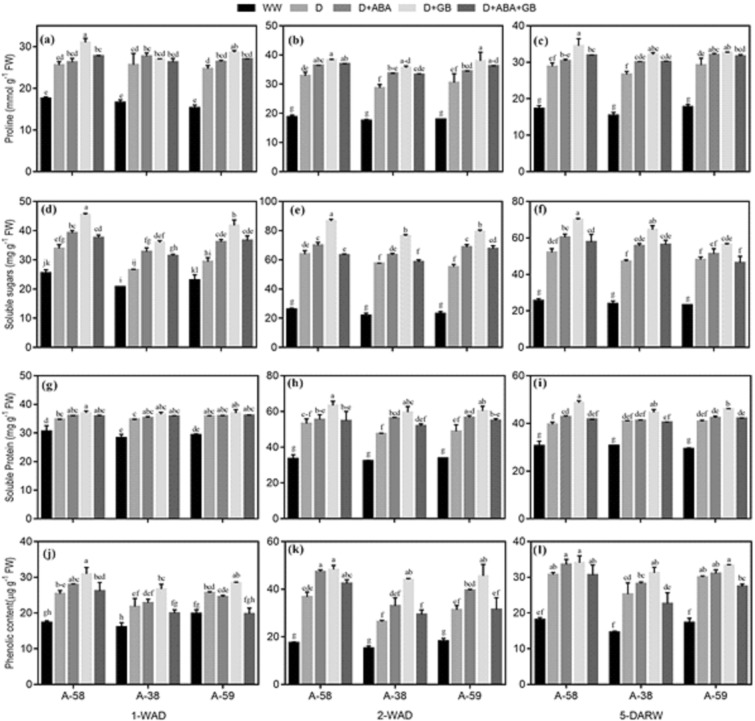
Effect of drought stress and subsequent re-watering on superoxide dismutase (SOD) (**a**–**c**), peroxidase (POD) (**d**–**f**), catalase (CAT) (**g**–**i**) and ascorbate peroxidase (APX) (**j**–**l**) in A-58, A-38 and A-59 accessions of *A. compressus*. The measurements were done at one week after drought (1-WAD), two weeks after drought (2-WAD) and five days after re-watering (5-DARW). Bars are mean ± SE of three replications (n = 3). The bars having the same letters don’t differ significantly at p ≤ 0.05.

The activity of antioxidant enzymes *viz*. SOD, CAT, POD and APX in all accessions of *A. compressus* was increased significantly due to drought stress. The activities of antioxidants were substantially higher in A-58 (4–143%) relative to A-38 (5–119%) and A-59 (19–112%) under drought stress, as compared to well-watered conditions which imparted it drought tolerance attribute. In addition, the activities of antioxidants were increased with time after exposure to drought stress (2-WAD > 1-WAD); whereas, declined again with recovery from drought stress (5-DARW). Nonetheless, application of ABA and GB alone or in combination further exaggerated the activities of antioxidant enzymes in all accession of *A. compressus* under drought stress and even after recovery from drought, as compared to untreated control. Maximum improvement in antioxidants activity in A-58 (11–67%, 20–49% and 6–44%), A-38 (14–67%, 11–61% and 13–26%) and A-59 (5–67%, 27–56% and 4–25%) was recorded by application of GB alone, 1-WAD, 2-WAD and 5-DARW, respectively (Fig. 5).

## Discussion

Drought enhanced the oxidative stress and lipid peroxidation manifested by increase in H_2_O_2_ and MDA contents in all accessions of *A. compressus* (Fig. 2). This increase lead to reduction in membrane stability (Fig. 2) and photosynthetic pigments (Fig. 3) during drought (1-WAD, 2-WAD) and recovery (5-DARW). However, application of ABA and GB alone or in combination mitigated the plants of all *A. compressus* accessions from drought induced damage by decreasing the ROS production and lipid peroxidation (Fig. 2); whereby, improving the membrane stability (Fig. 2) and photosynthetic pigments (Fig. 3) during drought and recovery. The decline in ROS production and lipid peroxidation in plants treated with ABA or GB was associated with improved osmolytes accumulation (Fig. 4) and activity of enzymatic antioxidants (Fig. 5) under drought. Previous studies have reported similar increase in osmolytes accumulation and antioxidants activity along with decrease in ROS production and increase in membrane stability under abiotic stress conditions^50,51^. Likewise, studies have reported that exogenous GB improved leaf water, osmotic and turgor potential, and osmolytes and enzymatic antioxidants activity which ultimately improved crop productivity^35,37^.

In present study, the effect of ABA and GB in improving osmolytes accumulation and antioxidants activity in *A. compressus* plants under drought stress was sustained over time during drought (1-WAD, 2-WAD) and even after recovery (5-DARW) (Figs. 4 and 5). Accumulation of osmolytes, and enhanced antioxidants activity are crucial stress tolerance mechanisms which are sustained over time in stress tolerant plants for better performance under drought stress and recovery in order to withstand the recurrent stresses^52,53^. It has been reported that plants retain the stress memory by accumulation of transcription factors for osmolytes and antioxidants even after recovery from stress in order to attain tolerance within no time when subjected to subsequent stress^9,54,55^. Moreover, plant growth regulators improve the stress tolerance and plant performance over time under stressed conditions and even in next progeny^20,30^. Previous studies have reported improved stress tolerance mechanisms by different plant growth regulators including ABA under chilling stress and after recovery^56,57^.

In present study, exogenous application of ABA and GB up-regulated the production and accumulation of proline, soluble proteins and sugars, and total soluble phenolics in all accessions of *A. compressus* under drought and after recovery (Fig. 4). Osmolytes accumulation improves the tissue water status through osmotic adjustment and quench the ROS in plants under stressed conditions^11^. Phenolic compounds contain aromatic ring in their structures which protects cellular membranes by ROS scavenging in plants under abiotic stresses^58,59^. Soluble proteins improves the hydration of cellular membranes and safeguards against oxidative damage of organic molecules under stress conditions^60^. Similarly, proline and soluble sugars aids in improving membrane stability by better water status attained by osmotic adjustment and quenching ROS^9,18,20^.

Drought stress increased the ROS production and lipid peroxidation in all *A. compressus* accessions; however, ABA and GB treated plants had better protected membranes (Fig. 2) and photosynthetic pigments (Fig. 3) owing to improved antioxidants system (Fig. 5). Plants have evolved an effective defense mechanism to quench ROS and confront against oxidative stress. It include both enzymatic and non-enzymatic defense system enabling plants to retain redox-balance^18^. SOD, one of the major antioxidant enzyme, detoxify the O^2−^ to O_2_ and ultimately to H_2_O_2_^61^. Later on, H_2_O_2_ is further reduced to H_2_O through the pursuits of CAT and POD^31,52,62^. Improved reaction rates of POD and APX under drought conditions exhibit the important operating-system of glutathione-ascorbate cycle to clean-up ROS^63^. In present study, enhanced activity of SOD, CAT, POD and APX in ABA and GB treated plants indicated the better defense mechanism against drought induced oxidative stress. Previous studies have similar increase in antioxidants by application of ABA^64^ and GB^65^ under drought stress.

All the accessions of *A. compressus* showed increase in ROS production and decrease in membrane stability (Fig. 2) and photosynthetic pigments (Fig. 3) in response to drought stress (1-WAD and 2-WAD) as well as recovery period (5-DARW). Nevertheless the deleterious effects of drought were more pronounced in A-38 while A-58 was found to be most tolerant. The maximum stress tolerance in A-58 was associated with enhanced accumulation of osmolytes (Fig. 4) and activity of antioxidants (Fig. 5) with concomitant decrease in H_2_O_2_ and MDA accumulation (Fig. 2). Moreover, ABA and GB application further improved the stress tolerance mechanisms in A-58 (Figs. 4 and 5). Production and accumulation of osmolytes, and activity of antioxidants is enhanced under drought stress which may be used as an index to measure the stress tolerance in plants^20^. Previous studies have also reported that accumulation of osmolytes and activity of antioxidants is greater in tolerant genotypes than sensitive ones^9,18^.

## Conclusion

Drought stress increased ROS production, lipid peroxidation, osmolytes accumulation and antioxidants activity in all accessions of *A. compressus*. The A-38 was the most sensitive while A-58 was the most tolerant among all accessions. Moreover, exogenous application of ABA and GB improved osmolytes accumulation, antioxidants activity and photosynthetic activity while decreased ROS and lipid peroxidation in all accessions of *A. compressus* during drought and recovery periods. Application of GB alone proved most effective in improving the drought tolerance mechanisms.
